# Idiopathic anterior mediastinal lymphangioma in a young adult: a rare case report and review of management

**DOI:** 10.1097/RC9.0000000000000390

**Published:** 2026-03-31

**Authors:** Ahmed Dawood Al Mahrizi, Fatima Mossolem, Bryan Gregory, Harman Gill, Adam Kaplan, Jackson Rejendran

**Affiliations:** a Faculty of Medicine and Surgery, University of Malta, Msida, Malta; b Praxis Institute, Voorhees, NJ, USA; c Department of Critical Care Medicine, Robert Wood Johnson Barnabas Health, NJ, USA

**Keywords:** anterior mediastinum, idiopathic, mediastinal lymphangioma, mediastinal mass, robotic-assisted surgery, robotic thoracic surgery

## Abstract

**Introduction and importance:**

Mediastinal lymphangiomas are rare benign malformations, comprising less than 1% of adult mediastinal tumors. Idiopathic cases in the anterior mediastinum are exceptional in young adults and often mimic malignancies like lymphoma, posing significant diagnostic challenges.

**Case presentation:**

A 24-year-old male presented with chest pain and B-symptoms (fever, night sweats). Imaging revealed a 7.5 cm well-circumscribed anterior mediastinal mass. Biopsy confirmed idiopathic lymphangioma. The patient underwent robotic-assisted thoracoscopic resection. Intraoperative findings showed pericardial involvement and compression of the superior vena cava and pulmonary artery. Complete excision was achieved without complications. Symptoms resolved immediately, and 3-month follow-up showed no recurrence.

**Clinical discussion:**

Differentiation from the “four T’s” (thymoma, teratoma, thyroid, lymphoma) requires histopathology. Although benign, these lesions compress vital structures. Robotic surgery offers superior visualization for precise dissection around major vessels compared to open thoracotomy, facilitating complete resection, critical to preventing recurrence (10–50% risk if incomplete).

**Conclusion:**

Robotic-assisted surgery is a safe short-term option for resecting rare idiopathic anterior mediastinal lymphangiomas. Early biopsy and complete excision aid in excluding malignancy and lowering recurrence risk, with longer-term follow-up essential.

## Introduction

The differential diagnosis for anterior mediastinal masses classically includes the “four T’s”: thymoma, teratoma, thyroid tissue, and the “terrible” lymphoma, which account for over 95% of cases[1]. Anterior mediastinal tumors arise in the anterior compartment, located between the sternum and pericardium[1]. Diagnosis is challenging due to the diverse etiologies, including neoplastic and non-neoplastic lesions[1]. These tumors often originate from thymic, lymphoid, or germ cell tissues, explaining their higher prevalence in children[1].

HIGHLIGHTS

- Idiopathic anterior mediastinal lymphangioma in a young adult is very rare.
- The case mimicked malignancy and was diagnosed via biopsy.
- Minimally invasive robotic surgery achieved complete mass removal.
- Patient recovered rapidly with no recurrence at 3 months follow-up.
- Early biopsy and minimally invasive management optimize patient outcomes.

One mass that has very rarely been reported in the anterior mediastinum is a lymphangioma[2]. Lymphangiomas are benign malformations of lymphatic vessels, predominantly congenital and seen in children[3]. Lymphangiomas in the adult population usually result from disruption of normal lymphatic drainage due to surgery, radiation, trauma, or previous malignancies[3]. Although most lymphangiomas occur above the diaphragm, mediastinal involvement is rare, comprising less than 1% of cases, particularly in acquired adult forms[4]. In cases that have presented in the mediastinum, it has been well-reported that they were either within the pediatric population or had inciting risk factors[4].

Treatment depends on the extent of invasion; well-circumscribed lesions have the best outcomes with surgical excision[5]. Surgical excision is typically the primary treatment modality for these tumors in both adults and pediatric populations[6]. While most are benign, neoadjuvant radiation may benefit lesions with malignant potential before excision[7]. Prognosis for these tumors is typically good especially with benign lesions, and a worse prognosis occurs in metastatic disease and with increased age[7].

We report a rare idiopathic anterior mediastinal lymphangioma in a young adult without predisposing factors. This case report has been reported in line with the SCARE checklist[8].

## Case presentation

### Patient information

A 24-year-old male with no significant past medical history presented to the emergency department with a 3-day history of chest pain, dry cough, intermittent fevers, night sweats, and decreased appetite. No current medications or allergies. Family history was negative for malignancy or lymphatic disorders. Social history: nonsmoker, occasional alcohol (<5 units/week), no illicit drugs. Review of systems: negative except for constitutional symptoms (fever, sweats, anorexia). He denied any history of trauma, surgery, radiation, or malignancy. Vital signs on admission included temperature of 38.2°C, heart rate 92 bpm, respiratory rate 18 breaths/min, blood pressure 118/76 mmHg, and oxygen saturation 98% on room air.

The operator was a board-certified cardiothoracic surgeon with 30 years of experience, including over 100 robotic thoracic procedures. Perioperative optimization included preoperative carbohydrate loading, multimodal analgesia (acetaminophen, gabapentin, and non-steroidal anti-inflammatory drugs), and early mobilization protocols per enhanced recovery after surgery guidelines. No patient perspective was formally documented, consistent with optional SCARE 2025 item 3 g; however, the patient verbalized satisfaction with rapid symptom resolution and cosmesis.

### Clinical findings

Physical examination revealed mild tachypnea and tachycardia but no lymphadenopathy, jugular venous distension, or focal neurological deficits. Chest auscultation revealed clear breath sounds bilaterally with no wheezes, rhonchi, or decreased air entry. Percussion was resonant throughout, without dullness suggestive of consolidation or effusion. The patient reported B-symptoms (fever, sweats, appetite loss) suggestive of possible lymphoma, though no weight loss was noted.

### Timeline

Day 0 (presentation): emergent evaluation for acute chest pain and associated symptoms.

Day 1: initial imaging (chest X-ray) performed; contrast-enhanced computed tomography (CT) of the chest ordered.

Day 3: CT-guided biopsy completed; pathology results pending.

Day 7: cardiothoracic surgery consultation; robotic-assisted procedure scheduled.

Day 10: surgical excision performed.

Day 14: postoperative recovery; discharge with resolved symptoms.

3 Months postoperatively: follow-up imaging (Table 1).

**Table 1:** Timeline of events.

Event	Date/Interval	Key Actions/Findings
Symptom Onset	3 days prior to ED visit	Chest pain, cough, fevers, sweats, anorexia
ED Presentation	Day 0	Stable vitals; B-symptoms noted
Initial Imaging	Day 1	Chest X-ray: large right hemithorax opacity
Advanced Imaging	Day 1	CT: 7.5 × 5.2 × 6.8 cm anterior mediastinal mass with fat stranding
Biopsy	Day 3	CT-guided; pathology: idiopathic lymphangioma
Surgery	Day 10	Robotic-assisted thoracoscopic resection
Discharge	Day 14	Symptom resolution; no adjuvant therapy
Follow-up	3 months	CT: complete resection, no recurrence

### Diagnostic assessment

**Figure 1.**
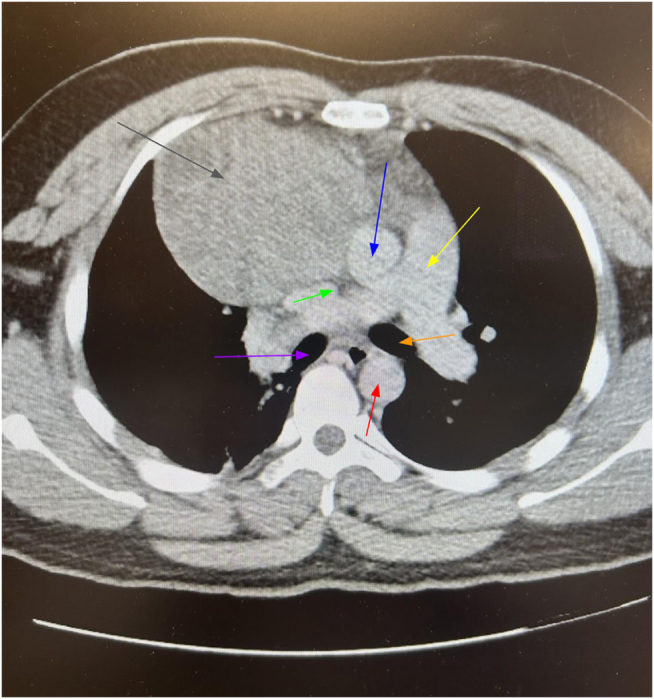
Axial CT scan of the chest (mediastinal window) showing a well-encapsulated, heterogeneous anterior mediastinal mass measuring approximately 7.5 cm × 5.2 cm × 6.8 cm. Gray arrow: mediastinal mass; Green arrow: superior vena cava; Blue arrow: ascending aorta; Red arrow: descending aorta; Yellow arrow: pulmonary artery trunk. The mass closely abuts the brachiocephalic vein, superior vena cava, ascending aorta, and right heart border without definitive invasion. Extension to the diaphragm and superior mediastinum is present. No pericardial or pleural effusion. Findings consistent with idiopathic lymphangioma. CT, computed tomography.

**Figure 2.**
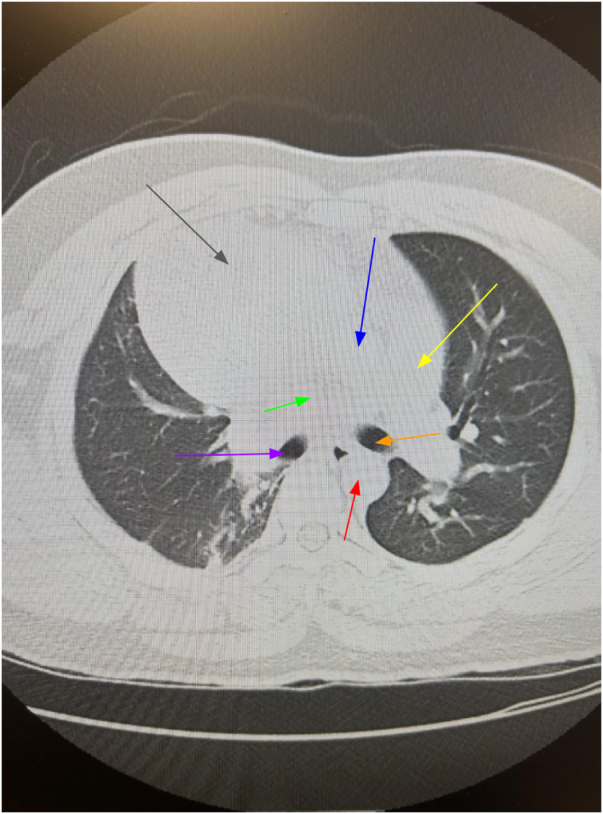
Axial CT scan of the chest (lung window) demonstrating a well-circumscribed, heterogeneous anterior mediastinal mass measuring approximately 7.5 cm × 5.2 cm × 6.8 cm. Gray arrow: mediastinal mass; Green arrow: superior vena cava compression; Blue arrow: ascending aorta; Red arrow: descending aorta; Yellow arrow: pulmonary artery trunk; Purple arrow: right mainstem bronchus; Orange arrow: left mainstem bronchus. The lungs show no focal airspace infiltrates, pleural effusion, or pneumothorax. No osseous or chest wall abnormality. The mass abuts but does not invade adjacent vessels or the heart, consistent with mediastinal lymphangioma. CT, computed tomography.

A chest X-ray demonstrated a large soft tissue opacity overlying the right hemithorax. Contrast-enhanced CT of the chest revealed a well-circumscribed 7.5 cm × 5.2 cm × 6.8 cm mass in the anterior mediastinum with surrounding fat stranding but no evidence of invasion. Given the B-symptoms and mass characteristics, trans-thoracic CT-guided core biopsy (posterior approach via the right paravertebral route) was performed under local anesthesia without complications such as pneumothorax, bleeding, or infection. Histopathology showed dilated lymphatic channels lined by benign endothelium (no atypia or mitoses), diagnostic of idiopathic lymphangioma (Figs. 1 and 2). Positron emission tomography–computed tomography (PET-CT) was deferred due to definitive benign biopsy and the low fluorodeoxyglucose-avidity expected in lymphangioma. Magnetic resonance imaging (MRI) was not pursued as contrast CT adequately characterized the cystic nature and vascular relationships for surgical planning. Angiography was omitted in the absence of vascular invasion on CT.

### Therapeutic interventions

Cardiothoracic surgery was consulted for definitive management. The patient underwent robotic-assisted thoracoscopic resection of the mass. The patient was positioned in the right lateral decubitus position at 45 degrees. The da Vinci Xi robotic platform (Intuitive Surgical) was used with four arms: a 30-degree scope via an 8 mm camera port (7th intercostal space mid-axillary), robotic instruments via 8 mm ports (5th/6th intercostal posterior axillary), and a assistant 12 mm port (4th intercostal anterior axillary). Intraoperative findings confirmed partial encasement without vascular injury; meticulous blunt/sharp dissection released adhesions. No vascular repair or grafts were required as the adventitia was preserved, ensuring complete excision (R0 margins). Intraoperatively, the mass showed pericardial involvement, compression of the superior vena cava, and partial encasement of the right main pulmonary artery, necessitating meticulous dissection for complete excision. No intraoperative complications occurred beyond these adhesions; the procedure was completed robotically without conversion to open surgery. Pathology of the resected specimen confirmed a benign idiopathic lymphangioma with negative margins.

### Follow-up and outcomes

Postoperatively, the patient’s constitutional symptoms (chest pain, cough, fevers, sweats, and anorexia) resolved dramatically within 48 hours. He was discharged on postoperative day 4 without the need for chemotherapy or radiation therapy. Follow-up CT imaging at 3 months confirmed complete resection of the mass with no evidence of recurrence or complications. The patient remains asymptomatic at the last follow-up.

No perioperative or postoperative complications occurred, aligning with expected outcomes for benign, well-circumscribed lesions. Planned surveillance includes CT imaging at 6 and 12 months, with annual clinical follow-up thereafter.

Written informed consent was obtained from the patient for publication of this case report and accompanying images.

## Discussion

Mediastinal lymphangiomas are rare benign vascular malformations originating from lymphatic tissue, accounting for less than 1% of all mediastinal tumors in adults. These lesions are predominantly congenital and most commonly diagnosed in pediatric populations, with approximately 90% manifesting by the second year of life. In adults, they are exceptionally uncommon and often acquired, typically resulting from disruptions in lymphatic drainage secondary to trauma, surgery, radiation, or prior malignancies. The presented case is particularly noteworthy as it involves an idiopathic anterior mediastinal lymphangioma in a young adult without identifiable risk factors, highlighting the potential for spontaneous occurrence even in this demographic^[^2,9,10^]^. Literature review reveals fewer than 20 adult mediastinal lymphangioma cases, predominantly managed via open surgery or video-assisted thoracoscopic surgery (VATS); robotic approaches are limited to pediatric or non-anterior cases. For instance, Fokkema *et al*. reported open resection for an adherent adult mediastinal lymphangioma, while recent VATS cases emphasize cystic variants without vascular encasement[2]. Our robotic success in an anterior lesion with superior vena cava (SVC) and pulmonary artery involvement adds to this sparse data, with only isolated reports of robotic feasibility in similar rarities.

The anterior mediastinum is the most frequent site for mediastinal lymphangiomas when they do occur, though posterior or cervicomediastinal extensions have also been reported. Clinical presentation is often nonspecific, with symptoms arising from mass effect or compression of adjacent structures, such as chest pain, cough, dyspnea, or constitutional symptoms like fevers and sweats, as seen in our patient. Imaging plays a crucial role in diagnosis; chest X-rays may reveal opacities, while contrast-enhanced CT typically demonstrates a heterogeneous, multiloculated cystic mass, as observed here with dimensions of 7.5 × 5.2 × 6.8 cm. MRI can further characterize the lesion as T2-hyperintense and multiseptated, aiding in differentiation from other cystic entities. Definitive diagnosis, however, requires histopathological confirmation, which in this case revealed features consistent with a congenital lymphangioma following CT-guided biopsy^[^2,9–13^]^.

The differential diagnosis of anterior mediastinal masses classically includes the “four T’s” – thymoma, thyroid tissue, teratoma, and lymphoma – which comprise over 95% of cases. Thymoma was excluded by the absence of calcification or enhancement patterns on CT and a negative biopsy for thymic epithelium. Teratoma was unlikely due to the homogeneous cystic appearance without fat or calcifications. Lymphoma was ruled out by benign endothelium without lymphoid atypia. Bronchogenic or pericardial cysts were differentiated by anterior location and vascular compression. Less common entities such as lymphangioma, bronchogenic cysts, pericardial cysts, or neurogenic tumors should be considered, especially in atypical presentations. In our patient, the presence of B-symptoms initially raised concern for lymphoma, emphasizing the importance of biopsy to rule out malignancy. Unlike malignant lesions, lymphangiomas lack invasive growth but can complicate surgically due to adherence to vital structures, as evidenced by the pericardial involvement and vascular encasement in this case.

Surgical excision remains the cornerstone of treatment for mediastinal lymphangiomas, offering curative potential when complete resection is achieved. Approaches vary from minimally invasive techniques like VATS or robotic-assisted procedures, as utilized here due to superior 3D visualization, tremor filtration, and seven degrees of freedom, enabling precise dissection around vascular structures like the SVC and pulmonary artery – advantages over VATS for complex encasements, with comparable safety but potentially shorter hospital stays per comparative studies[14]. Open thoracotomy was avoided given the well-circumscribed nature and the absence of invasion. Incomplete resection carries a recurrence risk of 10-50%, while radical excision is associated with excellent long-term outcomes and low recurrence rates. Adjuvant therapies such as sclerotherapy, radiotherapy, or chemotherapy have been explored for unresectable or recurrent cases but show variable efficacy and are not routinely recommended for benign lesions. In this instance, robotic-assisted excision led to complete resection, rapid symptom resolution, and no need for further interventions, aligning with reported prognoses of favorable outcomes in benign cases without metastasis.

Longer-term surveillance is planned, as incomplete resection carries 10-50% recurrence risk in lymphangiomas per series like the Mayo Clinic’s 25 cases[14].

This case emphasizes the value of maintaining a broad differential beyond the traditional “four T’s” for anterior mediastinal masses, particularly in young adults with idiopathic presentations. Early recognition through multimodal imaging and biopsy can facilitate timely surgical intervention, minimizing complications from compression of critical structures like the superior vena cava or pulmonary arteries. Providers should consider lymphangioma in similar scenarios to optimize management and improve patient outcomes.

### Strengths and limitations

Strengths

- Documents a rare idiopathic adult anterior mediastinal lymphangioma managed robotically, expanding the limited literature on minimally invasive approaches.
- Provides comprehensive timeline and imaging for educational value.
- Demonstrates complete resection with short-term success, informing surgical decision-making.

Limitations

- Short follow-up duration limits long-term recurrence data.
- Single-case design precludes generalizability.
- No molecular analysis of lymphatic malformation etiology.

## Conclusion

This case describes a rare idiopathic anterior mediastinal lymphangioma in a young adult, managed with robotic-assisted thoracoscopic resection that achieved complete excision (R0 margins) and rapid symptom resolution without adjuvant therapy. Biopsy proved crucial to confirm a benign etiology amid B-symptoms and rule out the classic four Ts (thymoma, teratoma, thyroid tissue, lymphoma). Minimally invasive robotic approaches enable precise dissection for well-circumscribed lesions near vascular structures, potentially aiding short-term success while planned surveillance (e.g., imaging at 6 and 12 months and annually) monitors for recurrence.

## Data Availability

All data generated or analyzed during this case report are included in this published article or its supplementary material (including the case presentation, imaging descriptions, and SCARE checklist). Raw imaging files are available from the corresponding author on reasonable request, subject to patient confidentiality and institutional policies.
